# The Uterus and Morphinomania

**Published:** 1887-12

**Authors:** 


### *THE UTERUS AND MORPHINOMANIA.*

Dr. Lutaud recently presented to *La Societé de Medicine* a valuable paper on this subject, of which the following is the summary:

"I would especially recommend the use of morphine to combat the hemorrhage of uterine cancer. In treating these cases, I have observed the relation of this therapeutic agent to the healthy and diseased womb. In the former case it is injurious, causing the menstrual functions to disappear, but in the latter it is useful, diminishing uterine activity, and reducing the hemorrhage and pain which accompany cancer and fibroma uteri. These facts may be considered as established:

"1. Chronic morphinism diminishes or suppresses menstruation.

"2. From this fact we are justified in giving morphine in uterine diseases which are aggravated by the menstrual flow, and which are not susceptible to curative treatment.

"In cancer of the uterus, I would recommend the production of the morphine habit, giving the drug subcutaneously, beginning with a quarter of a grain, and increasing the dose until from two to eight grains are taken during the day. In this manner the pain and hemorrhage are reduced to a minimum, a necessary stimulant is furnished and life is prolonged."—*Revue de Therapeutique.*

